# Warming Events Advance or Delay Spring Phenology by Affecting Bud Dormancy Depth in Trees

**DOI:** 10.3389/fpls.2020.00856

**Published:** 2020-06-19

**Authors:** Andrey V. Malyshev

**Affiliations:** Experimental Plant Ecology, University of Greifswald, Greifswald, Germany

**Keywords:** extreme warming events, warm pulses, dormancy induction, dormancy release, dormancy level, bud burst

## Abstract

The frequency of sudden, strong warming events is projected to increase in the future. The effects of such events on spring phenology of trees might depend on their timing because spring warming has generally been shown to advance spring budburst while fall and winter warming have been shown to delay spring phenology. To understand the mechanism behind timing-specific warming effects on spring phenology, I simulated warming events during fall, mid-winter and at the end of winter and quantified their effects on bud dormancy depth and subsequently on spring leaf out. The warming events were carried out in climate chambers on tree seedlings of *Betula pendula* and *Fagus sylvatica* in October, January, and February. Control seedlings were kept at photoperiod and temperature matching the daily fluctuating field conditions. Warmed seedlings were kept 10°C warmer than the control seedlings for 10 days during the respective warming periods. Warming in October increased bud dormancy depth and decreased spring leaf-out rate only for *F. sylvatica*, whereas warming in February reduced bud dormancy depth and advanced spring leaf-out rate only for *B. pendula*. Neither bud dormancy depth nor spring leaf out rate were affected by January warming. The results indicate that warming-induced changes in bud dormancy depth may explain species- and timing-specific warming effects on spring phenology. The extent to which the timing of bud dormancy phases is species-specific will influence among-species variation in future spring leaf out times.

## Introduction

Climate warming has often been associated with advanced spring phenology, not only via observations (Wesołowski and Rowiński, 2006; Wood et al., 2006; Richardson et al., 2010; Zohner and Renner, 2014), but also through experiments (Hole, 2014) and modeling studies (Luedeling et al., 2013; Lange et al., 2016). Recently, however, a slowing down of spring phenology advancement has been documented (Fu et al., 2015). Furthermore, temperature increases have been shown to both advance and delay budburst dates, depending on their timing (Heide, 2003; Fu et al., 2012; Luedeling et al., 2013). Antagonistic temperature affects on spring phenology may arise when the process of bud dormancy is variably affected by temperature changes, depending on the phase that the process is in when a temperature increase takes place.

Winter dormancy is an adaptation of perennial plants to survive seasonal unfavorable conditions by suspending growth and reducing their activity to a minimum. Dormancy is a state that buds of many temperate plant species develop which needs to be broken by a cold period in order for budburst to occur in spring (Sogaard et al., 2008). Dormancy depth is the level of dormancy at a particular time and is commonly estimated by exposing twig cuttings from trees to optimum growing conditions (Panchen et al., 2014; Vitasse et al., 2014). The amount of warmth required, often measured in growing degree days (GDD), also termed forcing requirements, approximates the dormancy depth. Experiments have shown that the forcing requirement increases exponentially with decreasing time spent at cool temperatures, generally accepted to be below 10°C, while being species-specific (Battey, 2000; Cesaraccio et al., 2004; Harrington et al., 2010). Furthermore, as bud dormancy depth is increased from late summer and throughout the fall, a process known as bud dormancy induction, warmer temperatures can further increase bud dormancy depth or delay its induction (Heide, 2003; Laube et al., 2014; Pagter et al., 2015). Delayed and/or deeper bud dormancy could require a longer cold period to bring the forcing requirements to the same level as in years with cooler fall temperatures. Absence of the additionally required chilling period, often driven by winter warm spells, means buds have higher bud dormancy depth in the spring and in turn open later under similar spring temperatures (Heide, 2003; Søgaard et al., 2009). Experiments showing how bud dormancy depth is affected by warming in different tree species and how such dormancy changes affect spring phenology are lacking.

In contrast to the potential delaying effect of fall warming on spring phenology (Heide, 2003), warmer temperatures can advance spring budburst if they occur after bud dormancy has largely been released following a cold period. At this time buds are in the ecodormancy phase, where cold temperatures have only a minor effect on further reducing bud dormancy depth and warm temperatures have a stronger ability to reduce bud dormancy depth by fulfilling the forcing requirements and thus advancing budburst (Kramer, 1994). There is also evidence that early flushing species, such as *Betula pendula*, require shorter chilling periods to lower their bud dormancy depth than late successional species, such as *Fagus sylvatica*, which often require a longer cold period before warm temperatures advance bud break (Murray et al., 1989; Zohner and Renner, 2014). Furthermore, in *F. sylvatica*, short photoperiod additionally prevents rapid dormancy release to a larger extent as in other tree species (Vitasse and Basler, 2013; Malyshev et al., 2018), resulting in reduced sensitivity to warming periods earlier in the year when day length is still short. It is also possible that the timing of bud dormancy induction is different in *B. pendula* compared with *F. sylvatica*, further suggesting that the same warming at a particular period may increase bud dormancy depth and hence delay budburst in one species while having a lesser effect on the other.

The effects of short-term, sudden warming events during different bud dormancy phases on changes in bud dormancy depth and subsequent spring budburst dates are unclear. Heat waves are predicted to increase in frequency and duration under future climate scenarios (Schär et al., 2004; IPCC, 2014) and future phenology models need to account for the potentially different impacts of warming during different bud dormancy stages. Furthermore, the effects of strong warming events on typically early and late flushing species need to be studied to quantify whether budburst advances or delays are likely to be more pronounced in either tree group.

I selected a common early flushing and a common late flushing tree species to study the effects of sudden strong warming events on spring phenology. I subjected tree seedlings to either fall, midwinter or end of winter warming events inside climate chambers, each time increasing the temperature by 10°C relative to ambient temperature in the field. I estimated bud dormancy depth of the seedlings prior to and after the warming events. Spring phenology after each warming event was subsequently recorded. I hypothesized that warming would delay spring phenology of seedlings in which the dormancy depth had increased following warming and vice versa.

## Materials and Methods

### Plant Material

Tree seedlings were grown from a local seed source, stemming from northern Germany. One-year-old birch *Betula pendula* seedlings and two-year-old *Fagus silvatica* seedlings were grown in a local tree nursery and potted in April 2016 in 3 L pots with sandy loam soil. Seedlings were 40–70 cm tall. Fertilization was limited to horn shavings for slow release of nutrients and watered weekly. In June 2016 the trees were delivered to the test field site, located in Greifswald Germany. Direct sunlight was reduced by a net, stretched 2 m above the trees, reducing radiation by about 30%. In September the pots were placed in pre-dug holes in a sand area for additional insulation against frost. In total, 75 seedlings were used per species. Prior to each warming event, four seedlings per species were sampled destructively to estimate the starting dormancy depth. During each warming event, nine seedlings per species were placed in a warming chamber and nine seedlings per species were placed in a control chamber. Four of the nine seedlings were sampled destructively after the warming events while five seedlings were returned to the field site. Seven samplings were kept at the field site for the duration of the experiment to represent ambient budburst in the spring.

### Simulation of Warming Pulses

**FIGURE 1:**
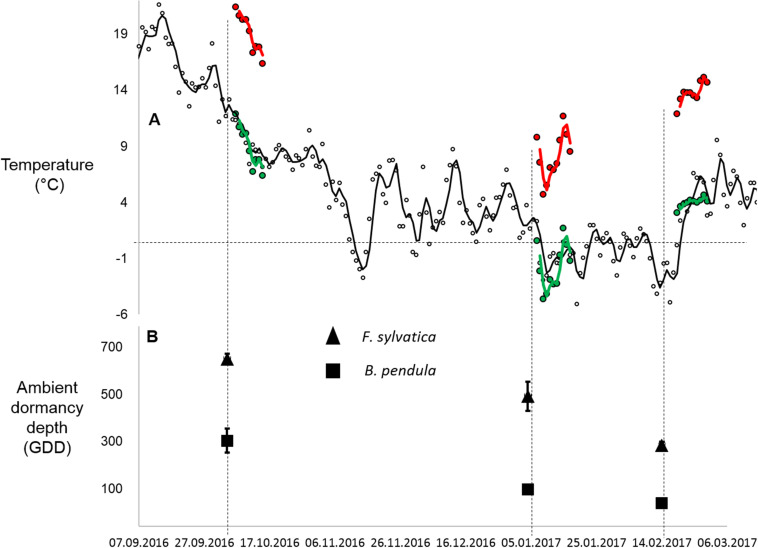
**(A)** Daily mean temperatures of the warming and control chamber temperatures (warming in red; control in green) during each simulated warming pulse. Ambient temperatures at the field site where tree seedlings were overwintered are in black. Lines indicate 2-day running means. **(B)** Estimated dormancy depth of tree seedlings at the beginning of each warming pulse. Dormancy depth was estimated by calculating growing degree days (GDD) of twig cuttings prepared from ambient tree seedlings under optimum growing conditions in climate chambers. Triangles stand for *B. Pendula* and squares stand for *F. sylvatica* (*n* = 8–12 per sampling date per species). Error bars indicate standard error.

Warming was simulated for a period of 10 days during three periods, starting on 4.10.2016, 4.1.2017, and 14.2.2017. Warming was simulated in climate chambers (Model: LT-36VL, CLF Plant Climatics GmbH, Germany). One climate chamber was programmed to keep temperature and daylight changes as close to ambient conditions as possible, adjusting its photoperiod and temperature daily (Figure 1 and Table 1). In the warmed chamber temperature was maintained 10°C above the control chamber temperature. The warming magnitude was selected to reflect the biggest differences in mean January air temperatures for the Mecklenburg Vorpommern region of Germany in the last 50 years (local weather data). The duration of 10 days was chosen to reflect data that warming for 6 days at 9°C during the non-growing season can be substantial enough to cause differences in plant growth (Malyshev et al., 2016). To avoid chamber bias, seedlings and chamber settings were switched between the two chambers every 2 days. The actual warming and control chamber temperatures were 19/9°C, 8/−2°C and 13/4°C for the respective warming periods. Standard deviations were similar between treatments and ambient temperature, with treatment standard deviations being between 0.4 and 0.9°C higher in the chambers compared to ambient temperatures (Table 1). Mean humidity inside the control and warmed chambers varied between 70 and 90%, with the differences between the control and warming chambers ranging between 1 and 5% during the warming periods. Humidity was allowed to covary with temperature manipulation. At the conclusion of warming, five trees were returned to the field site while four trees were destructively sampled to estimate bud dormancy depth of each tree after the treatment.

**TABLE 1:** Temperature means and standard deviation of temperatures in the control and warming chambers as well as the ambient temperature at the field site during each temperature manipulation period.

**Warming period**	**Treatment**	**Mean temp (°C)**	**Standard deviation**	**GDD sum**	**Chilling day sum**
4th–14th November 2016	Ambient	9.2	1.5	38	0
	Control chamber	10.0	1.9	35	0
	Warming chamber	19.1	1.7	127	0
4th–14th Janaury 2017	Ambient	–0.8	1.6	0	10
	Control chamber	–1.8	2.2	0	10
	Warming chamber	8.1	2.2	34	1
14th–24th February 2017	Ambient	3.6	3.0	5	7
	Control chamber	4.1	3.4	0	9
	Warming chamber	13.8	3.8	88	0

*Daily sums of growing degree days (GDD) and chilling day sums (number of days where temperature was equal to or below 5°C) for each period are also presented.*

### Effect of Warming on Dormancy Depth

Additional four tree seedlings of each species were destructively sampled before and four after each warming event to estimate the change in bud dormancy depth due to warming. Bud dormancy depth was estimated by making five twig cuttings from each of the four seedlings. Each twig cutting was approximately 8 cm in length and had at least 2 lateral buds. Seedlings had four to eight branches from which four were selected from different vertical tree sections to obtain a representative sample of the average dormancy of all lateral buds on the tree. Top portions of branches were used to create the cuttings. The terminal bud was removed and candle wax was used to prevent desiccation from the top cut surface. Removal of the terminal bud ensures that true dormancy of lateral buds can be observed without the influence of terminal buds which can prevent lateral buds from opening in the fall via paradormancy (Champagnat, 1989). Using twig cuttings to describe bud dormancy changes was used instead of using whole tree seedlings due to space limitation and the method having been deemed appropriate previously (Vitasse et al., 2014; Primack et al., 2015), having been widely used to track bud dormancy changes (Ghelardini et al., 2009; Zohner and Renner, 2015; Vitra et al., 2017). The twigs were placed in deionized water in a climate chamber with a 24 h photoperiod (PAR 60–100 μmol m^–2^ s^–1^) and a temperature of 22°C (± 2°C). The twigs were inserted into a Polyethylene foam 1 cm thick which floated in trays of water. Twigs were recut under water weekly and water was changed twice a week. Mean days to budburst for the first three twigs (out of five) per tree were recorded to estimate the time to achieve approximately 50% budburst as not all buds opened on all sampled dates for all species (*n* = 12 per treatment and species). As minor temperature differences existed during the dormancy tests, the number of days to budburst was converted to GDD, commonly used to quantify dormancy depth and calculated as:

$$G\,D\,D=\sum_{t_{0}}^{t_{1}}\left(T_{m\,e\,a\,n}-T_{b\,a\,s\,e}\right)$$

where t_0_ is the starting day of the warming period, t_1_ is the day at which budburst is observed, T_mean_ is the daily mean temperature, and T_base_ is a constant (5°C), representing a minimum temperature threshold required for stimulating budburst (Polgar and Primack, 2011; Fu et al., 2016). The warming effect on dormancy depth was calculated by dividing the dormancy depth of each twig (GDD) of each seedling following warming by the mean dormancy depth of all seedlings prior to warming (a ratio of dormancy depth after/before warming). Resulting values above 1 represented an increase in dormancy depth and values below 1 represented a decrease in dormancy depth following treatment with respect to initial dormancy depth. Dormancy depth at the start of each warming event for each species is shown in Figure 1. For *F. sylvatica*, only the first twig which had bud bust in each tree seedling was used for the all twigs from the October and January treatments due to high twig mortality (resulting *n* = 4 per treatment).

### Phenology Monitoring

Fall leaf coloration differences were measured 2 weeks after the conclusion of the first warming event to estimate if dormancy differences were accompanied by differences in senescence rates. Six random leaves were measured per tree with a SPAD device (SPAD-502 Plus, Konica Minolta, Inc.).

Spring phenology was recorded via two methods. Firstly, percent budbreak was recorded for each tree every 2 days to estimate the date of 50% bud break. Data for *Fagus sylvatica* was lost for this first method. Secondly, the lengths of six most unfolded leaves on each tree was measured as soon as all trees from any treatment group had reached 90% complete leaf unfolding. Early flushing species often take much longer to achieve full leaf unfolding compared with late flushing species, meaning that a long delay/advancement in budburst dates may be reduced to only minor differences in the number of days with respect to completely unfolded leaves, which is most important in gaining a photosynthetic advantage. Furthermore, differences in dates of full leaf unfolding are also largely dependant on the temperature at the time of unfolding. Thus, the unfolding stage of leaves was numerically quantified by measuring the length from the emerging leaf tip to the bud. Choosing the day of measurement when the seedlings from the earliest flushing treatment group had 90% of their leaves completely unfolded approximated the maximum difference in leaf out stages among the treatment groups and made these differences better comparable between species. To make between-species comparison even more robust and better represent treatment effects on leaf unfolding, each leaf length was divided by the mean leaf length of untreated ambient tree seedlings. Seven ambient seedlings per species were measured.

### Statistical Analysis

The effect of warming on leaf coloration after the first warming event was tested via an ANOVA where species and treatment (control/warming) were the influencing factors and leaf coloration (SPAD values) was the response variable.

The effect of warming on dormancy depth was evaluated by a three-way ANOVA, where the timing of warming, species and treatment (control/warming) were the influencing factors and dormancy depth ratio was the response variable. Individual tree identity was included as a random factor in the linear mixed model. Dormancy depth ratios were log-transformed to improve the homo- genetic of variances and the normality of residuals.

The effect of warming on the date of 50% budburst was analyzed for *Betula pendula* with a two-way ANOVA, where the timing of warming and treatment (control/warming) were the influencing factors and the date on which 50% budburst occurred for each tree was the response variable.

Transformation of ∧0.5 was applied for the leaf ratios to improve the homogeneity of variances and the normality of residuals. All analyses were performed using R statistical software (R Core Team, 2013). R packages lme4 and lmerTest were used for the ANOVA analyses while the package emmeans was used to compare the effects of treatments within each species and warming periods.

## Results

### Leaf Coloration After Fall Warming

**FIGURE 2:**
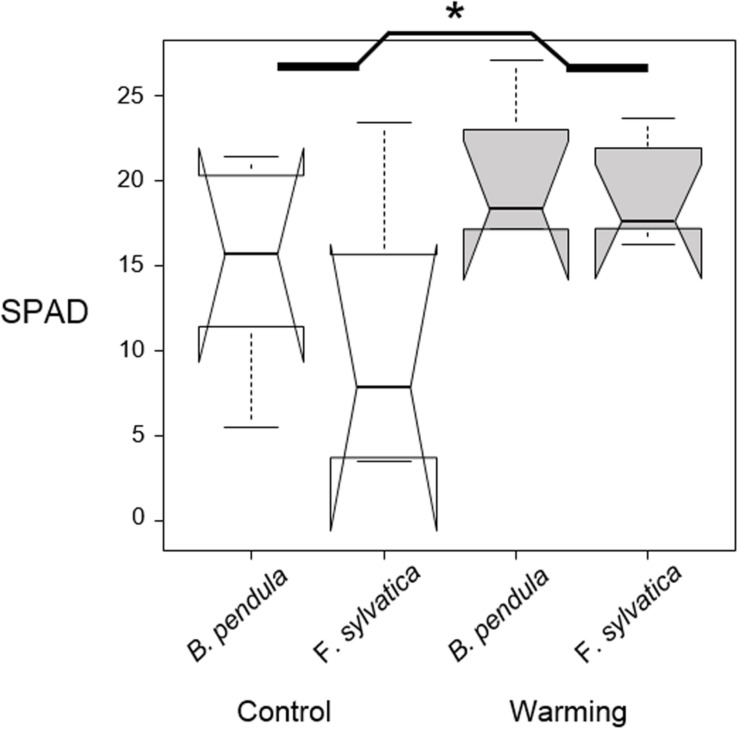
Median SPAD values of six random leaves per tree seedling, measured 2 weeks after the end of the first warming pulse in October. No interaction between species and treatment was found. Significant difference between warmed and control plants is shown with the asterisk. Notches show the 95% confidence interval of the median and whiskers extend to a maximum of 1.5 × IQR beyond the box.

October warming delayed leaf senescence of warmed seedlings compared with the control plants, with no interaction having been detected between treatment and species (Table 2 and Figure 2). Seedlings were approximately 30% more green after warming compared with control seedlings (*p* = 0.04).

**TABLE 2:** Influence of species (*B. pendula* and *F. sylvatica*), treatment (control/warming), and timing of warming (4th October, 4th January, and 14th February) on four response variables, as affected by increased temperature of 10°C for 10 days.

**Response variable**	**Factor (s)**	***F*-value**	***p*-value**
SPAD	Species	0.4	0.54
	**Treatment**	**5.1**	**0.04**
	Species* Treatment	0.7	0.41
Change in dormancy depth relative to ambient seedlings	Timing	2.8	0.07
	**Species**	**5.7**	**0.02**
	Treatment	0.2	0.63
	Species* Timing	**16.1**	**<0.001**
	Species* Treatment	**16.0**	**<0.001**
	Timing * Treatment	**7.6**	**<0.01**
	Species* Timing *Treatment	2.5	0.095
Change in spring mean leaf length relative to ambient seedlings	**Timing**	**106**	**<0.001**
	Species	0.18	0.68
	**Treatment**	**8.3**	**0.006**
	Species* Timing	**6.4**	**0.004**
	Species* Treatment	**4.2**	**0.05**
	Timing * Treatment	0.03	0.97
	Species* Timing *Treatment	**6.2**	**0.004**
Date of 50% budburst in spring	**Timing**	**37.0**	**<0.001**
	**Treatment**	**18.8**	**<0.001**
	Timing * Treatment	**19.7**	**<0.001**

*SPAD response was only measured after the fall warming event. Date of 50% budburst was only measured in B. pendula in the spring. Bold text signifies significant effects of respective factors.*

### Relative Change in Bud Dormancy Depth After Warming

**FIGURE 3:**
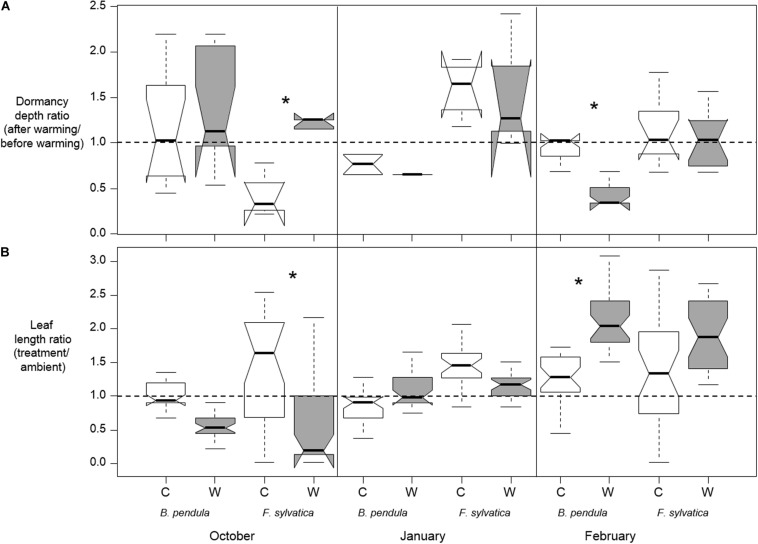
**(A)** medians of bud dormancy depth ratios for each treatment, species and timing of warming (10 days of + 10°C). C stands for the control treatment and W stands for the warming treatment (see section “Materials and Methods”). Dormancy depth was estimated at the start and end of each warming pulse via twig cuttings (*n* = 4–12 per treatment per species per warming date), calculating the growing degree days required for bud break. Four tree seedlings were destructively sampled prior to and after each warming event. **(B)** Medians of leaf length ratios (treatment leaf length/mean leaf length of ambient tree seedlings) of each treatment and species measured in the spring following warming in October, January, and February. Leaf lengths were measured in 6 biggest leaves on each tree seedling (5 trees per treatment) when all tree seedlings of the earliest flushing treatment group had 90% completely unfolded leaves. Five tree seedlings were used per treatment (control/warming) per date per species while seven tree seedlings were kept under ambient conditions. Significant differences between treatments are shown with asterisks. Notches show the 95% confidence interval of the median and whiskers extend to a maximum of 1.5 × IQR beyond the box.

The effect of warming on bud dormancy depth depended on the timing of warming as well as being species-specific (*p* < 0.001 for Species^∗^Treatment interaction and *p* < 0.01 for Timing^∗^Treatment interaction; Table 2 and Figure 3A). In October, a greater increase in dormancy depth occurred in warmed seedlings than in control seedlings only for *F. sylvatica* (*p* < 0.001). No effect of warming was detected in January for either species and in February a greater decrease in dormancy depth occurred in warmed seedlings than in control seedlings only for *B. pendula* (*p* < 0.001). In *F. sylvatica* dormancy depth was increased by approximately 3 times after fall warming compared to control, whereas the dormancy depth of *B. pendula* was decreased by approximately 2.5 times after February warming compared to control.

### Effect of Warming on Day of 50% Budburst in *B. pendula*

**FIGURE 4:**
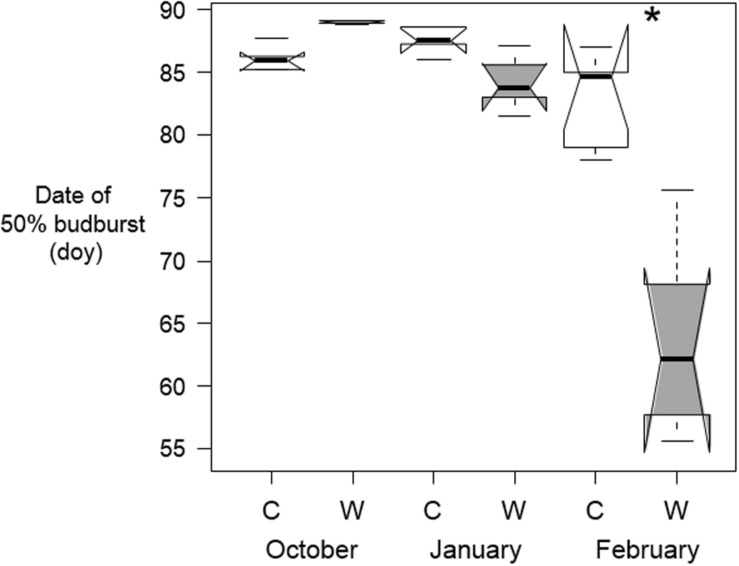
Median dates on which 50% of buds on tree seedlings of *B. pendula* opened in the spring after being warmed by 10°C (W) and the control trees (C) during October, January, or February (*n* = 5 per treatment and sampling date). Significant differences between treatments are shown with asterisks. Notches show the 95% confidence interval of the median and whiskers extend to a maximum of 1.5 × IQR beyond the box.

The effect if warming on budburst dates for *B. pendula* was timing-specific (*p* < 0.001 for Timing^∗^Treatment interaction). The date of 50% budburst in *B. pendula* was only affected by February warming, whereby warmed tree seedlings opened their buds 3 weeks earlier than the control seedlings (Table 2 and Figure 4). Warming at other times had no effect on budburst dates.

### Relative Change in Leaf Lengths Following Warming

There was a three-way interaction between the influence of treatment, species and timing of treatments on the relative change in leaf length in the spring (*p* = 0.004 for the Species^∗^ Timing ^∗^Treatment interaction; Table 2 and Figure 3B). The effect of warming on leaf length ratios thus depended on the timing of warming as well as being species-specific. The three-way interaction resulted from warming tending to increase the leaf ratio in *B. pendula* after January warming while tending to decrease it for *F. sylvatica* (Figure 3B). In October, a greater decrease in leaf ratio occurred in warmed seedlings than in control seedlings only for *F. sylvatica* (*p* < 0.001). No significant effect of warming was detected in January for either species and in February a greater increase in leaf ratio occurred in warmed seedlings than in control seedlings only for *B. pendula* (*p* = 0.05). In *F. sylvatica* leaf length decreased by approximately 6 times after fall warming compared to control, whereas leaf length *B. pendula* increased by approximately 1.6 times after warming compared to control in end of winter warming.

## Discussion

The antagonistic ability of warming to both delay and advance spring phenology has rarely been mechanistically explained. I have shown that the delaying effect can occur when bud dormancy is increased following warming and the advancing effect happens when warming reduces bud dormancy. Whether bud dormancy depth is increased or decreased following warming likely depends on the dormancy phase of buds, with warming during the induction phase likely increasing it and warming during the ecodormancy phase decreasing it. Furthermore, the timing of dormancy induction and ecodormancy seem to be species-specific, explaining why warming at a particular period may increase the dormancy depth in one species, but not in another.

**FIGURE 5:**
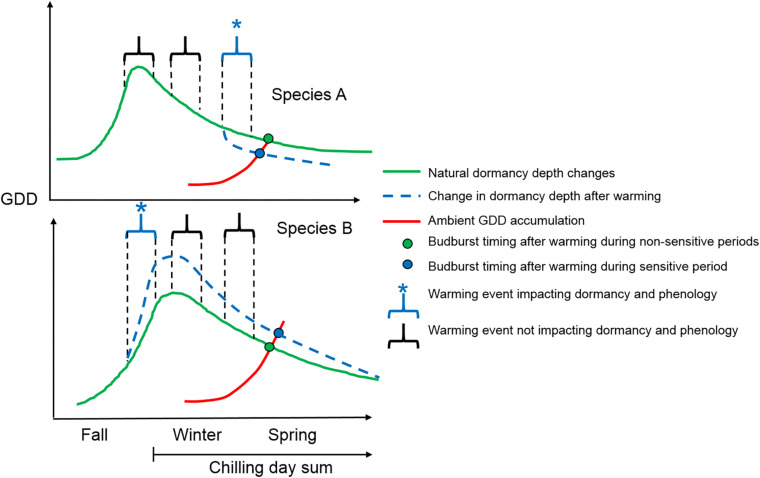
Conceptual visualization showing how warming of the same magnitude (relative to ambient temperature) can have different effects on bud dormancy depth, depending on its timing and species. Species A represents a typically early flushing species (such as *B. pendula*) and Species B represents a typically late flushing species (such as *F. sylvatica*). The *y*-axis represents theoretical bud dormancy depths at different stages of bud dormancy induction and release phases in contrasting species (only indicative of species-specific temporal changes in dormancy depth). Species A has an earlier bud dormancy induction phase, not reacting to the first fall warming event during which the dormancy induction phase has been completed. In species B the fall warming increases bud dormancy depth (blue line), which results in a delay in spring budburst (blue point vs. green point). The spring budburst occurs when the accumulation of growing degree days (GDD – red line) from the date of maximum dormancy depth reaches the threshold GDD requirement at the end of winter (intersection of red and blue/green lines). The second warming event does not affect dormancy depth of any species as the dormancy depth is still very high and the additional GDD from warming are counterbalanced by the reduction in dormancy due to more chilling day accumulation in the absence of warming. The last warming event at the end of winter reduces bud dormancy depth in Species A (blue line), the dormancy depth of which has almost stagnated and become non-responsive to further chilling day accumulation. As a consequence, species A flushes earlier in the spring following the end of winter warming event (blue point vs. green point). Bud dormancy of Species B is still high at this point and chilling day accumulation is able to reduce dormancy depth as fast as the additional GDD from the warming event.

Bud dormancy was increased by warming earlier in the season in *F. sylvatica* when the dormancy process was likely in its induction phase (Figure 5). The increase in bud dormancy depth has previously been tracked in several trees and found to take place in the fall, ending (reaching peak bud dormancy) between October and December (Boyer and South, 1989; Champagnat, 1989; Calmé et al., 1994). In the fall, attaining a deeper dormancy depth during the period of dormancy induction with increased temperature has been shown in poplar (Kalcsits et al., 2009) and maple (Westergaard and Eriksen, 1997). The reason behind warmer temperature increasing dormancy depth remains to be unknown. A warming-induced delaying effect on spring phenology during dormancy induction has also been shown, both experimentally (Heide, 2003), and retrospectively using modeling approaches explaining warming effects on spring phenology with historical climate and budburst dates (Roberts et al., 2015). Thus, warming during the fall season is likely to continue acting as an antagonist to the advancing effect of spring warming on spring phenology, albeit not to the same extent in all species. No warming effect on bud dormancy depth for *B. pendula* can potentially be explained by a different timing of bud dormancy induction in these species, although evidence for this is lacking. Another late successional species, *Quercus rubra*, has been shown to end its bud dormancy induction later than another birch species (*Betula alleghaniensis*) (Calmé et al., 1994). *B. pendula* may experience bud dormancy induction earlier in the season compared with *F. sylvatica*, already having attained full endodormancy (peak dormancy depth) before the October warming event. Regardless of the reason, future bud dormancy changes following warming in the fall are shown here to be species-specific and need to be modeled accordingly. Previous modeling approaches have suggested a stronger delaying effect of fall warming on spring phenology for *Betula pendula* compared with *Fagus sylvatica* (Roberts et al., 2015), disagreeing with my results. The discrepancy may be due to minor temperature increases in the modeling study, likely confounded with continued warming throughout the non-growing season. Experiments are thus needed that simulate a gradient of warming levels from bud dormancy induction to its release to show how projected warming will affect bud dormancy changes and in turn spring phenology in different tree species.

Bud dormancy was not affected in either species following mid-winter warming as warming took place during a period when dormancy depth was still high in both species (Figure 5). After attaining full dormancy, the optimal chilling temperature for fastest dormancy reduction is thought to be around 5°C or below (Murray et al., 1989; Heide, 2003; Junttila and Hanninen, 2012; Vitasse and Basler, 2013). The control treatment had 10 chilling days while the warming seedlings experienced only 1 chilling day. Trees from the warming treatment did accumulate more GDD, however (34 vs. 0 GDD), likely compensating for the reduced dormancy depth of the control seedlings. Both increased GDD and chilling days can reduce dormancy depth, with the effectiveness of chilling days being much stronger the higher the initial dormancy depth (Myking and Heide, 1995). Therefore, the seedlings’ bud dormancy could have been reduced by the same amount via more chilling days in the control group and by higher accumulation of GDD in the warming treatment. Subsequently, both treatments resulted in a similar bud dormancy depth at the end of the treatment, leading to similar spring bud burst dates. The same increase in temperature in winter may therefore have milder effects on spring phenology compared with fall and end of winter or spring warming events.

Late winter warming occurred after dormancy depth has naturally already been decreased by the accumulation of chilling days at ambient field conditions, at least in *B. pendula* (Figure 5). Furthermore, much higher accumulation of GDD occurred in the warming treatment compared with the control (88 vs. 0.) Even though nine more chilling days had accumulated in the control treatment, likely having reduced bud dormancy, the rate at which bud dormancy is reduced via chilling during this time is much lower compared to periods when dormancy depth is much higher, as previously shown in other studies (Caffarra and Donnelly, 2010; Harrington et al., 2010; Malyshev et al., 2018). *F. sylvatica* did not react to late winter warming, potentially because its dormancy depth was still high at the initiation of the warming treatment. The additional chilling days in the control treatment were thus likely just as effective in decreasing the still high dormancy depth in *F. sylvatica* as the higher number of GDD in the warming treatment. Many studies have documented the high chilling requirements required to reduce dormancy depth in *F. sylvatica* compared with other tree species (Murray et al., 1989; Zohner and Renner, 2014; Malyshev et al., 2018), driven additionally by short photoperiod additionally reducing the rate of dormancy decrease in the species (Heide, 1993; Vitasse and Basler, 2013; Malyshev et al., 2018). Pioneer species such as *B. pendula*, which require few chilling days to release their dormancy (Heide, 2003), may therefore react more sensitively to future early spring warming periods, advancing their spring phenology to a greater extent.

In both species, fall warming delayed fall leaf coloration, yet only increased bud dormancy depth in *F. Sylvatica*. Studying and modeling dormancy induction (its timing and depth) with respect to future temperature increase may thus be more beneficial in predicting future spring phenology changes than merely monitoring senescence dates. An increased dormancy depth following warming may be the underlying cause behind the observed delayed spring phenology following delayed autumn senescence (Heide, 2003; Fu et al., 2016) and may explain the lack of phenological responses to warming in certain plant species (Cook et al., 2012). An extended growing season in the fall may have variable spring phenology knock down effects, due to its species-specific and timing –specific effects on bud dormancy. Still, milder warming, acting over months rather than days as tested here, may affect dormancy changes differently (Ex., shifting the dormancy induction timing rather than increasing dormancy depth) and needs to be further studied.

Years with strong warming spells may experience several warming spells throughout the year. Therefore, a year with a strong fall warming event may also be accompanied with winter and/or spring warming spells. Therefore, the cumulative effect of several warming spells throughout the year, including the unexplored effect of summer warming, needs to be evaluated further with future similar experiments. Effects of single extreme warming pulses on dormancy and spring phenology in species with contrasting seasonal bud dormancy patterns can be summarized, however, as seen in Figure 5. Nonetheless, the effects of milder and more prolonged warming (longer than 10 days) on dormancy depth and spring phenology have not been addressed here and need to be studied. Furthermore, the delaying effect of fall warming on spring phenology may be offset by the increased heat accumulation later on in the non-growing season (Fu et al., 2019). Additionally, within-species variation in warming induced bud dormancy changes has not been quantified here, although evidence shows that strong spring phenology and bud dormancy differences exist in both species studied here (Falusi and Calamassi, 1996; Junttila and Hanninen, 2012; Robson et al., 2013; Kramer et al., 2017) as well as in other species (Boyer and South, 1989). Lastly, bud dormancy depth prior and after warming events was measured in different trees here. Measuring bud dormancy depth changes in the same trees, with methods as least invasive as possible, will likely improve the correlation between the effects of temperature on bud dormancy and spring phenology.

The experiment has been carried out on tree seedlings and it is unclear if adult trees will behave the same way. Additionally, roots and buds likely experienced only mild temperature differences in the climate chambers. For mature trees, snow and leaf layer, combined with deep root growth can result in very different temperatures above and below ground (Sturm et al., 1997). Whether root temperature can affect bud dormancy is not known.

In conclusion, I have shown that differential effects of future extreme warming events on spring phenology will likely to depend on non-linear responses of bud dormancy depth to warming. Warming-induced changes in bud dormancy depth are likely to depend on the timing of warming events as well as on species-specific timing of bud dormancy induction and release. It is therefore necessary to experimentally track temporal changes in dormancy depth in different tree species from dormancy induction to its release to identify especially sensitive periods for each species.

## Data Availability Statement

The datasets generated for this study are available on request to the corresponding author.

## Author Contributions

AM carried out the experiment, analyzed the data, and wrote the manuscript.

## Conflict of Interest

The authors declare that the research was conducted in the absence of any commercial or financial relationships that could be construed as a potential conflict of interest.
